# Optimization of CNN through Novel Training Strategy for Visual Classification Problems

**DOI:** 10.3390/e20040290

**Published:** 2018-04-17

**Authors:** Sadaqat ur Rehman, Shanshan Tu, Obaid ur Rehman, Yongfeng Huang, Chathura M. Sarathchandra Magurawalage, Chin-Chen Chang

**Affiliations:** 1 Department of Electronic Engineering, Tsinghua University, Beijing 100084, China; 2 Faculty of Information Technology, Beijing University of Technology, Beijing 100022, China; 3 Department of Electrical Engineering, Sarhad University of Science and IT, Peshawar 25000, Pakistan; 4 School of Computer Science and Electronic Engineering, University of Essex, Colchester CO4 3SQ, UK; 5 Department of Information Engineering and Computer Science, Feng Chia University, Taichung City 407, Taiwan

**Keywords:** CNN optimization, image classification, MRPROP, training algorithm

## Abstract

The convolution neural network (CNN) has achieved state-of-the-art performance in many computer vision applications e.g., classification, recognition, detection, etc. However, the global optimization of CNN training is still a problem. Fast classification and training play a key role in the development of the CNN. We hypothesize that the smoother and optimized the training of a CNN goes, the more efficient the end result becomes. Therefore, in this paper, we implement a modified resilient backpropagation (MRPROP) algorithm to improve the convergence and efficiency of CNN training. Particularly, a tolerant band is introduced to avoid network overtraining, which is incorporated with the global best concept for weight updating criteria to allow the training algorithm of the CNN to optimize its weights more swiftly and precisely. For comparison, we present and analyze four different training algorithms for CNN along with MRPROP, i.e., resilient backpropagation (RPROP), Levenberg–Marquardt (LM), conjugate gradient (CG), and gradient descent with momentum (GDM). Experimental results showcase the merit of the proposed approach on a public face and skin dataset.

## 1. Introduction

The convolution neural network (CNN) algorithm is the most state-of-the-art algorithm due to its vast diversity of applications, which are found in many areas: classification, recognition, detection, etc. The strength of a CNN lies with its enormous capabilities of feature extraction in its layers, which are synchronized in a feed-forward manner. Furthermore, the training algorithms and output classification layer further boost up the performance. Due to the aforementioned characteristics, a deep CNN is suitable for many computer vision problems where the solutions are difficult to obtain analytically. CNN has solved many computer vision problems efficiently, e.g., handwritten digit recognition [1,2], optical character recognition [3], car detection [4], face detection [5,6], face recognition [7,8], and analysis of facial expression [9].

Previous studies have shown that many researchers have tried to improve the efficiency of the CNN through pretraining filter banks in supervised and unsupervised manners, and by adding more number of layers (deepness) [10]. Jarrett et al. [11] indicated that multi-stage feature extraction procedures produce results with better accuracy than single-stage feature extraction procedures. Also, filter learning (supervised, unsupervised, and random) boosts up the performance efficiency. Moreover, rectification and local contrast normalization also have a positive impact on a CNN in object recognition tasks. Erhan et al. [12] and Glorot et al. [13] suggest that the initialization of the weights and training algorithm directly affects the convergence rate and efficiency of a CNN. Particularly, training algorithms, whether supervised or unsupervised, are highly responsible, because it is difficult to train a network’s first few layers and weights from backpropagation due to vanishing/exploding gradients. Therefore, choosing a simple algorithm, which allows efficient training and optimized weight adjustment, is highly desirable. 

Calculation of the system error function gradient is also one of the important parts of the CNN training method. Most of them include complex computations; therefore, it may be difficult to meet real-time system requirements with such methods. The error function and the system error of the control application of neural networks are correlated [14], such as the error backpropagation algorithm (EBP) [15]. The convergence of the EBP algorithm is very slow if many gulches characterize the error function. An additional variable called “momentum” is added to the weight update in order to improve the training performance of the CNN [16]. A similar approach is proposed by Orlowska-Kowalska et al. [17], in which they attached a simple proportional derivative control to the gradient processing. This technique significantly increases the algorithm efficiency, as the gradient function is not computed directly.

The resilient backpropagation (RPROP) algorithm [18] is also independent of accurate gradient calculation. Also, a high computational proficiency and quick convergence speed are essential benefits. Due to the aforementioned properties, the RPROP algorithm is chosen for CNN [19] training for the face classification task. However, in RPROP, the continuous alterations of weights did not lead to any constant value; therefore, continuous deviations are enforced. As the continuous weights change in CNN, the training process is never-ending, which leads to system instability. Therefore, in this paper, we bring forward a modified training algorithm, modified resilient backpropagation (MRPROP) for the CNN [19], which helps the system by performing better weight changes to accomplish desired outputs efficiently and swiftly. Our contribution is as follows:

- We propose the tolerant band Δ*K* in MRPROP to avoid CNN overtraining.
- Global best concept is introduced in weight updating criteria for optimization.
- The MRPROP allows the CNN to optimize its weights more swiftly and precisely.

## 2. Resilient Backpropagation Algorithm (RPROP)

Training in CNN is considered to be the most important part of this work. RPROP is an efficient training algorithm that performs weight step alterations based on local gradient data. The key aim of choosing this training algorithm in CNN is that the weight adaptation is not blurred by gradient behavior at all. In addition, RPROP plays a significant role in optimizing the weights of the CNN due to the following properties.

1. The RPROP algorithm is fast, and requires less memory compared with other training algorithms.
2. The RPROP algorithm is independent of the shape of error surfaces [19].
3. The sign of error function gradient is utilized instead of the values of the error.

Let *del_xy_* be the individual update value of each weight between the *x*th neuron and the *y*th input at the “*t*” training step, which determine the size of the weight increase or decrease. The increase/decrease in the size of the weight is solely determined by the local sight of error function *E*(*t*), based on the below learning conditions of Equation (1):

(1) $$del_{xy}^{t}=\{\begin{array}{c} \rho^{+}\times \;del_{xy}^{t-1};if\;\frac{\partial E^{\left(t-1\right)}}{\partial w_{xy}}\times \;\frac{\partial E^{\left(t\right)}}{\partial w_{xy}}>\;0\\ \rho^{-}\times \;del_{xy}^{t-1};if\;\frac{\partial E^{\left(t-1\right)}}{\partial w_{xy}}\times \;\frac{\partial E^{\left(t\right)}}{\partial w_{xy}}<0\\ del_{xy}^{t-1};Elsewhere \end{array}$$

where $0<\rho^{-}<1$ and $\rho^{+}>1$.

Weight is adjusted in each iteration/epoch to make the actual output closer to the desired output. Therefore, in each iteration, the algorithm jumps to local minima, and the update value *del_xy_* is decreased by factor $\rho^{-}$, indicating that the last update is too big due to the change of the partial derivative of the corresponding weight *w_xy_* sign. However, if the derivative maintains its sign, the update value is slightly augmented to speed up the convergence, in order to escape from the local minima. Moreover, when the update value is adapted for each weight, the weight update occurs according to a very simple rule: the update value decreased the weight, in case the error is growing. Conversely, in the case of a negative derivative, the weight is increased by its update value, i.e., it is being added. This can be mathematically illustrated as:

(2) $$delw_{xy}^{t}=\{\begin{array}{c} -del_{xy}^{t};if\;\frac{\partial E^{\left(t-1\right)}}{\partial w_{xy}}>\;0\\ +del_{xy}^{t};if\;\frac{\partial E^{\left(t-1\right)}}{\partial w_{xy}}<0\\ 0\;;Elsewhere \end{array}$$

(3) $$w_{xy}^{t+1}=\;w_{xy}^{t}+delw_{xy}^{t}$$

where $delw_{xy}^{t}$ is the weight updated between the *x*th neuron and the *y*th input, at the “*t*” training step.

Nevertheless, if the partial derivative changes sign, i.e., the minimum was missed due to a too-large previous step, the previous update of the weight is reverted. Mathematically:

(4) $$delw_{xy}^{t}=-del_{xy}^{t-1};if\;\frac{\partial E^{\left(t-1\right)}}{\partial w_{xy}}\times \;\frac{\partial E^{\left(t\right)}}{\partial w_{xy}}<0$$

Therefore, due to this backtracking weight step, the derivative is supposed to alter its sign once more in the following step. So, to avoid this computational expense of the update value, there should be no adaptation in the succeeding step of the update value by setting $\frac{\partial E^{\left(t-1\right)}}{\partial w_{xy}}=0$ in the *del_xy_* adaptation rule above.

## 3. Modified Resilient Backpropagation Algorithm 

In this work, the convergence speed and performance of the RPROP algorithm is enhanced by a novel approach. Some modifications are made in MRPROP, so that it will reach and achieve global solutions faster than the basic RPROP algorithm.

The overall error is defined as the mean square error (mse) between the network outputs and the desired outputs; mathematically, it can be written as:

(5) $$E_{mse}=\frac{1}{U\times NL}\sum_{u=1}^{U}\sum_{n=1}^{NL}{\left|y_{n}^{U}-d_{n}^{U}\right|}^{2}$$

where the training set has a *U* input data matrix and *U* desired output representations; *X^U^* represents the *u*th training image; *d^U^* is our corresponding preferred output vector; and $y_{n}^{U}$ ynurepresents the actual network output. This shows the function of the overall network parameters i.e., weights and biases.

It is obvious that the error generated during the training process influences the output error function. This error is calculated at each step of the training process for a presumed set of weights. Taking this into consideration, the gradient of the CNN error function can be calculated as follows:

(6) $$f_{xy}\left(t\right)=\frac{\Delta E\left(t\right)}{\Delta W_{xy}\left(t\right)}=\;\frac{E\left(t\right)-E\left(t-1\right)}{W_{xy}\left(t\right)-W_{xy}\left(t-1\right)+\Delta z_{i}}$$

where *E*(*t*) and *E*(*t* −1) represent the error in training steps *t* and *t* − 1, respectively; and $W_{xy}\left(t\right)$ and $W_{xy}\left(t-1\right)$ are the weights between the *x*th neuron and the *y*th input in training steps *t* and *t* − 1. Whereas, $\Delta z_{i}$ is introduced to avoid division by zero, in case the weight changes halted, and for describing the sign of the slope, regardless of the inaccurate value of $f_{xy}\left(t\right)$.

When the analysis of the parameter *ρ* factor fluctuates in the RPROP algorithm, as determined by Equation (1), it can be observed that the condition of keeping a factor at a constant value is achieved only if:

(7) $$f_{xy}\left(t\right)\cdot f_{xy}\left(t-1\right)=0$$

This state practically does not exist in real case scenarios by any system processing physical measurement data. Therefore, the continuous alterations of the $del_{xy}$ factor do not lead to any constant value, but continuous deviations are enforced. Also, due to the continuous alteration in the CNN weights ($delw_{xy}$), it is impossible to achieve the state described by Equation (7). This leads to a never-ending training process. Due to this, CNN overtraining can be noticed, which leads to system instability. In order to avoid such a situation, a modification of the RPROP algorithm is proposed. We make two major modifications in the MRPROP algorithm. Firstly, we introduce a tolerant band Δ*K* into the training conditions of Equation (1). Secondly, a global best concept is incorporated into the weight updating criteria, in order to allow the training algorithm of CNN to optimize its weights more swiftly and precisely to find a good solution, as shown in Equations (8) and (9).

The introduction of Δ*K* helps to protect the system against overtraining, which in return affects the overall efficiency of CNN in a positive way. A properly selected range of the tolerant band Δ*K* is found through using the trial and error method after comprehensive tests on a wealth of case studies with various operating points, assuring the stable operation of the CNN network, and limiting the excessive increase in the MRPROP weights.

To obtain more reliable solutions for CNN weight updating and achieve an optimized solution swiftly, we propose the “global best” concept in weight updating criteria. In RPROP, the change in weight Δ*w* depends on whether the updated value *del_xy_* increased or decreased according to error, in order to reach a better solution. However, the previously updated values are neglected after every iteration; this means that all of the best values previously achieved in the weight changes would not be referred back. Hence, there is no information sharing between the optimized values that have been achieved at the previous iterations and the current result. Therefore, by using the term “global best” concept in MRPROP, the information of previous weight change is the only source for the accurate result. Therefore, the past optimized value is randomly selected from all of the updated values of the previous weight change, and is used to update the process. This variable is called global best, or “*gbst*”. For a minimization problem, the global best selection procedure is given as follows:

- First, select two updated values randomly from all of the past change in weights Δ*w*.
- Compare these two values against the optimized solution, and choose the better one as *gbst*.

Hence, the new proposed selection strategy will ensure that the diversity of the update value is preserved to avoid being trapped into a local optimum. The “*gbst*” is selected on the optimized value of current population delw, as shown in Equations (8) and (9):

(8) $$del_{xy}^{t}=\{\begin{array}{c} \min\;\left(\rho^{+}\times \;del_{xy}^{t-1},\;gbst\right);if\;f_{xy}\left(t\right)\cdot f_{xy}\left(t-1\right)>\;\Delta K\\ \max\;\left(\rho^{-}\times del_{xy}^{t-1},gbst\right);if\;f_{xy}\left(t\right)\cdot f_{xy}\left(t-1\right)<\;-\Delta K\\ del_{xy}^{t-1}\;;\;if-\Delta K\leq f_{xy}\left(t\right)\cdot f_{xy}\left(t-1\right)\leq \;\Delta K \end{array}$$

(9) $$delw_{xy}^{t}=\{\begin{array}{c} -del_{xy}^{t}\cdot \;sign\left(f_{xy}\left(t\right)\right);if\;f_{xy}\left(t\right)\cdot f_{xy}\left(t-1\right)>\;\Delta K\\ del_{xy}^{t}\cdot \;sign\left(f_{xy}\left(t\right)\right);if\;f_{xy}\left(t\right)\cdot f_{xy}\left(t-1\right)<\;-\Delta K\\ 0\;;\;if\;-\Delta K\leq f_{xy}\left(t\right)\cdot f_{xy}\left(t-1\right)\leq \;\Delta K \end{array}$$

**Figure 1:**
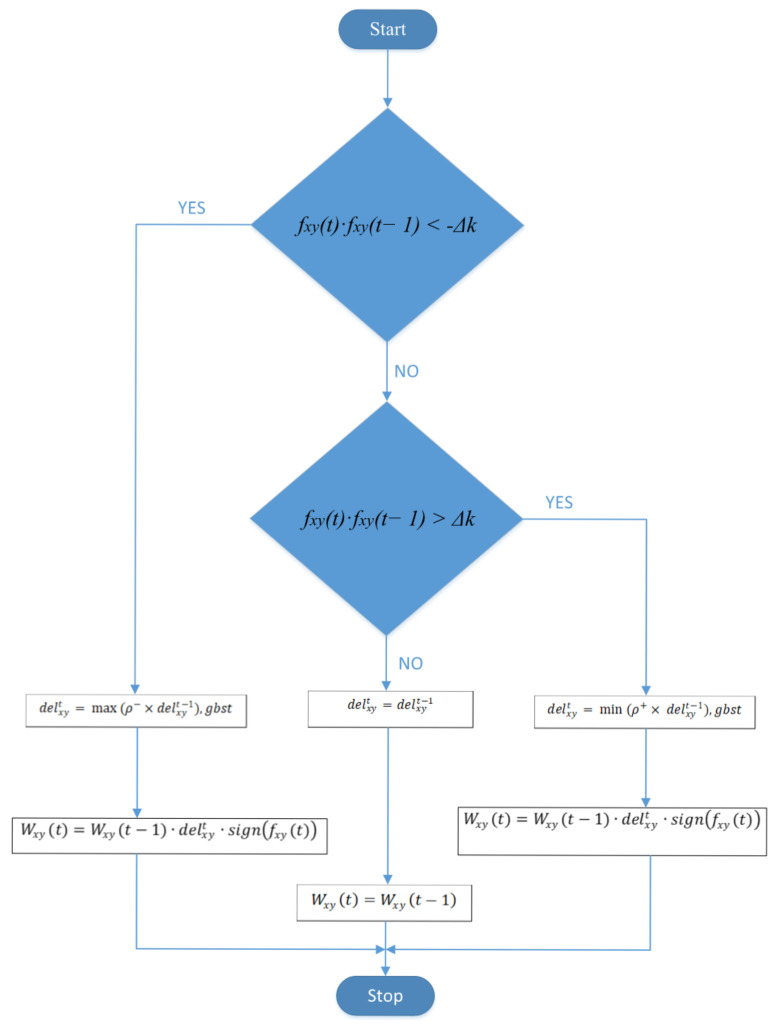
Flowchart of the proposed modified convolution neural network (CNN) training procedure through modified resilient backpropagation (MRPROP).

A number of satisfactory experimental tests are shown in Table 1, which prove the appropriate operation of the CNN with the proposed approach. The block diagram of the MRPROP for the CNN is shown in Figure 1. It is clear that the introduction of the tolerant band and the variable *gbst* cause not only the optimized weights that are achieved, but also the suboptimal weights. Similarly, the proposed strategy helps the system optimize its weight more swiftly and precisely, which directs the CNN into its best position and rapidly converges the training algorithm to its global optimum solution.

## 4. Experimental Results and Discussion

In this section, we analyze the convergence speed, output evaluations, training time, and number of training epochs based on the weight optimization criteria of the five different training algorithms. For comparison, in this article, we focus on five representative training algorithms, namely: resilient backpropagation (RPROP) [20], Levenberg–Marquardt (LM) [21], gradient descent with momentum (GDM) [22], conjugate gradient (CG) [23], and the proposed training algorithm (MRPROP). Our main objective is to determine a training algorithm for CNN that is fast, robust, and capable of handling large training datasets independent of the weights initialization strategy (supervised, unsupervised, random). The convergence speed of the training algorithm is highly affected by weight initialization and the choice of parameters that are used when training. For example, a slow decay in the mean square error (mse) occurs through a small learning rate α in the training algorithm; however, a large value of α may lead the training of the network to divergence. Subsequently, it is not possible to calculate all of the choices of the training parameters for CNN; we have determined the parameters by using the trial and error method after comprehensive mathematical experiments on a wealth of case studies. Yet, the optimized weight selection approach and training parameters in this paper reveal the basic tendencies in the convergence speed of the corresponding training algorithms for CNN.

**Figure 2:**
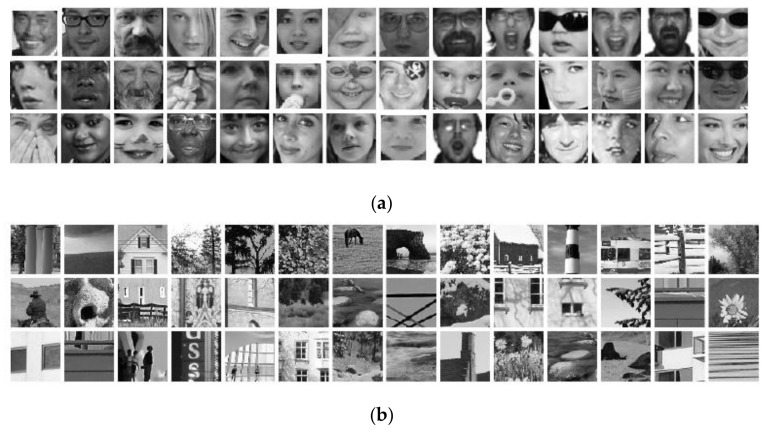
Sample images from Phung et al. [24] for evaluation of different training algorithms through CNN. (**a**) Face images; (**b**) Non-face images.

The four different training algorithms, along with the proposed training algorithm for a CNN, are assessed on a face/non-face classification problem. The dataset contains 20,000 images with 10,000 face images and 10,000 non-face images, which are manually cropped web images and randomly extracted scenery photos, respectively. A sample of the dataset is shown in Figure 2, and its statistics are depicted in Table 2.

The size of the input images was 20 × 20 pixels, which is similar to the image sizes that have been used by numerous authors for face/non-face classification [25,26,27]. The network was trained on an individual training algorithm for 2000 epochs. To compare the performance, we utilize ten-fold cross-validation of different indicators i.e., mse training, training time, the number of output evaluations, and the number of training epochs.

**Figure 3:**
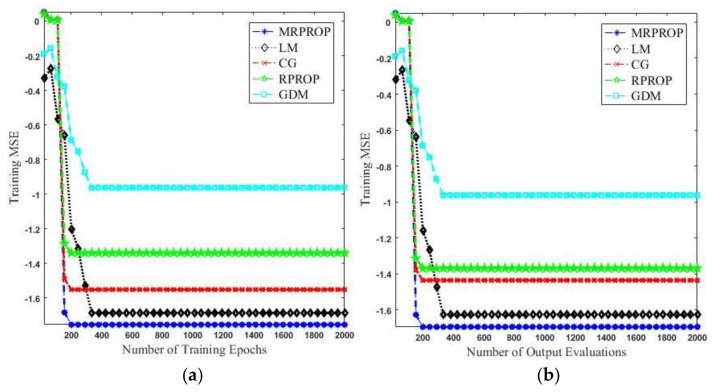

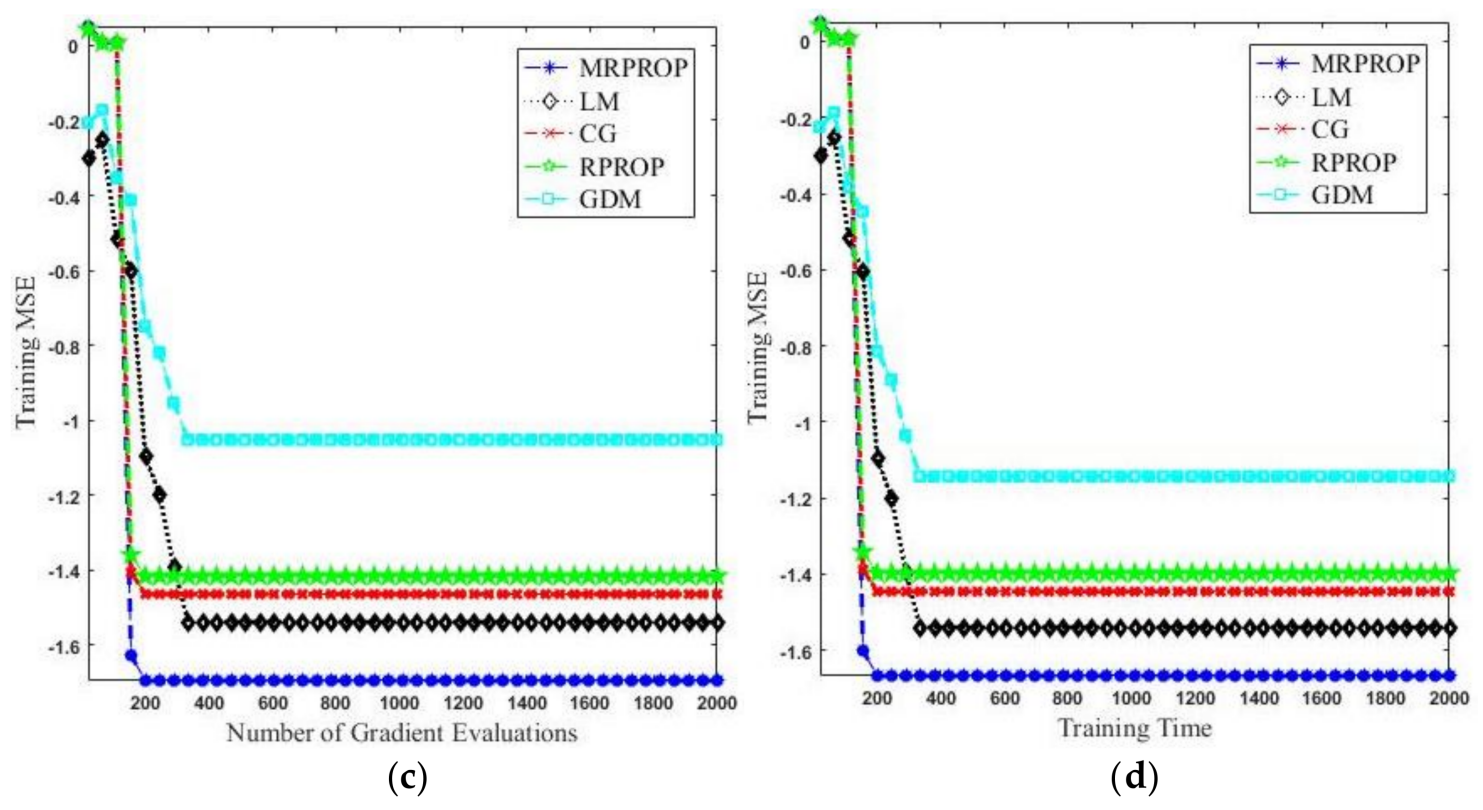
Comparison on face and skin dataset of five different training algorithms for CNN. mse training versus (**a**) total number of training epochs, (**b**) total number of output evaluations, (**c**) total number of gradient evaluations, and (**d**) total training time.

Figure 3 indicates the comparison results of CNN using five different training algorithms. It is clear from Figure 3a that for each epoch count, the proposed MRPROP attained the lowest mse among all of the other training algorithms of CNN, followed by LM. Meanwhile, GDM achieved the highest mse, followed by RPROP. Figure 3b shows that MRPROP took a smaller number of output evaluations to acquire a lower mse, compared to LM, CG, RPROP, and GDM. The CG and RPROP algorithms had a very similar performance, with a slight difference. 

Figure 3c reveals the number of gradient evaluations against the mse error function. It is clear from Figure 3c that our proposed algorithm takes a smaller number of gradient evaluations to achieve a lower mse. For example, the MRPROP algorithm uses 86 output evaluations to reach a mse of 0.35, whereas the LM and CG algorithms need 452 and 512 gradient evaluations, respectively. The RPROP algorithm has a better performance than the GDM algorithm when the number of output evaluations is above 200.

Figure 3d shows the training time of the individual training algorithms. In order to obtain a reliable comparison of the training speed, we measured the training time in terms of the MRPROP epoch time unit. We define one MRPROP epoch time unit as: the average time taken to perform one MRPROP training epoch on a fixed training set and a fixed-size network; it remains stable during the MRPROP training process. All four training algorithms—MRPROP, LM, CG, and RPROP—converged faster than the GDM algorithm. MRPROP was the fastest training algorithm among all of them due to its better weight optimization strategy compared to the other algorithms, which had no such strategy for weight regulations.

Table 1 depicts the classification accuracy of the CNN, with different training algorithms proposed for experimental purposes. It demonstrates that the CG and RPROP networks had similar classification rates, of 98% and 98.4% respectively, on the training dataset, and 96.9% and 97.1% on the testing dataset (the overall difference was no more than 0.4%), respectively, whereas the GDM achieved the lowest classification rates (97.1% and 96.2%). The highest classification rates were achieved by the proposed method, which were 99.2% and 97.8% on the training and testing dataset, respectively. These relative performances are consistent with the training speed comparison that was discussed earlier. CNN training with slower algorithms, such as GDM, requires more training time to find an optimal solution, as shown in Figure 3.

## 5. Conclusions

A new CNN training algorithm for visual classification problems, called MRPROP, has been presented. Features extraction at CNN layers is determined entirely through training. Our analysis of the five different training algorithms reveals that the RPROP and CG algorithms have reasonable convergence speeds and require small memory storage, whereas the LM algorithm is fast, but requires significantly more memory. However, the proposed MRPROP outperforms all of the other training algorithms, both in term of convergence speed and small memory usage. When evaluated on the face and skin dataset, CNN achieved classification rates of 99.2% and 97.8% with MRPROP on the training and testing datasets, respectively, which is significantly better than the same CNN with other training algorithms i.e., LM, CG, RPROP, and GDM. 

In future, we plan to apply this concept on a range of CNN problems to explore its competency in diverse application domains. We also intend to study possible extensions of the pretraining algorithms and their connected theoretical guarantees with CNN convergence, optimality, and efficiency. 

## Figures and Tables

**Table 1:** Classification Results of Different Training Algorithms on Face and Skin Dataset. GDM: gradient descent with momentum; RPROP: resilient backpropagation; CG: conjugate gradient; LM: Levenberg–Marquardt.

Training Algorithm	Efficiency on Training Dataset	Efficiency on Test Dataset
GDM	97.1%	96.2%
RPROP	98.4%	97.1%
CG	98.0%	96.9%
LM	98.6%	97.3%
MRPROP	99.2%	97.8%

**Table 2:** Statistics of Face and Skin Dataset [24].

Skin Type and Lightening Condition	Images
Whitish, pinkish	1665
Dark brown, reddish	965
Yellowish, light brown	1402
Other Skin type	102
Indoor lightening conditions	1931
Outdoor lightening conditions	1855
Other lightening conditions	214
